# Redundant Anaerobic Antimicrobial Prescriptions in German Acute Care Hospitals: Data from a National Point Prevalence Survey

**DOI:** 10.3390/antibiotics9060288

**Published:** 2020-05-28

**Authors:** Seven Johannes Sam Aghdassi, Petra Gastmeier, Michael Behnke, Sonja Hansen, Tobias Siegfried Kramer

**Affiliations:** 1 Charité–Universitätsmedizin Berlin, Corporate Member of Freie Universität Berlin, Humboldt-Universität zu Berlin, and Berlin Institute of Health, Institute of Hygiene and Environmental Medicine, Hindenburgdamm 27, 12203 Berlin, Germany; petra.gastmeier@charite.de (P.G.); michael.behnke@charite.de (M.B.); sonja.hansen@charite.de (S.H.); tobias.kramer@charite.de (T.S.K.); 2 National Reference Center for Surveillance of Nosocomial Infections, 12203 Berlin, Germany

**Keywords:** antimicrobial stewardship, anaerobic spectrum, antimicrobial overuse, point prevalence survey, Germany

## Abstract

Despite limited indications, redundant anaerobic antimicrobial prescriptions (RAAPs) are frequent. The objective of this study was to assess the prevalence and characteristics of RAAPs in German acute care hospitals. In a retrospective data analysis, antimicrobial prescriptions from a point prevalence survey on antimicrobial use in German acute care hospitals in 2016 were analyzed and RAAPs were identified. RAAPs were defined as a patient simultaneously receiving any of the following combinations: Penicillin/beta-lactamase inhibitor (PenBLI) plus clindamycin; PenBLI plus metronidazole; PenBLI plus moxifloxacin; PenBLI plus carbapenem; carbapenem plus clindamycin; carbapenem plus metronidazole; carbapenem plus moxifloxacin; clindamycin plus metronidazole; clindamycin plus moxifloxacin; and metronidazole plus moxifloxacin. Data from 64,412 patients in 218 hospitals were included. Overall, 4486 patients (7%) received two or more antimicrobials. In total, 441 RAAP combinations were identified. PenBLI plus metronidazole was the most common anaerobic combination (*N* = 166, 38%). The majority of RAAPs were for the treatment of community-acquired (*N* = 258, 59%) infections. Lower respiratory tract infections (*N* = 77; 20%) and skin/soft tissue infections (*N* = 76; 20%) were the most frequently recorded types of infections. RAAPs are common in German hospitals. Reducing redundant antimicrobial coverage should be a key component of future antimicrobial stewardship activities.

## 1. Introduction

Prescription of antimicrobials is associated with adverse events, such as toxicity, selection of multidrug-resistant bacteria, and *Clostridioides difficile* infection (CDI). Antimicrobials with anaerobic effectiveness, in particular, can have a detrimental effect on the human gut microbiome [1,2]. In addition, increasing rates of antimicrobial resistance in anaerobic bacteria have been observed [3,4]. However, prescription of anaerobic antimicrobials with inappropriate indication appears common [5]. Despite their similar effective ranges, medications, such as penicillin/beta-lactamase inhibitor (PenBLI), carbapenems, and moxifloxacin, are frequently combined with medications like metronidazole and clindamycin. There are only a few indications for which anaerobic antimicrobial combinations can be regarded as appropriate. Among these indications are the treatment of coinfections, such as CDI, with metronidazole, or the addition of clindamycin to the treatment of toxic shock syndromes [6]. However, the majority of redundant anaerobic antimicrobial prescriptions (RAAPs) have to be regarded as inappropriate [7]. The reduction of inappropriate prescribing is a primary goal of antimicrobial stewardship [8]. Few articles have documented the extent of redundant anaerobic coverage [7,9]. While some effective intervention strategies to reduce redundant anaerobic prescriptions have been reported [10,11], knowledge on the matter remains scarce. Point prevalence surveys (PPSs) can be a useful tool to collect data on antimicrobial prescriptions. Secondary analysis of PPS data can be a means to evaluate the quality of antibiotic prescription practices in the absence of days of therapy-based surveillance systems [12].

The primary objective of this study was to assess the extent of RAAPs in German acute care hospitals by analyzing data collected in a national PPS on healthcare-associated infections (HAIs) and antimicrobial use. Further objectives were to describe the most frequent combinations of anaerobic antimicrobials, and to describe the most common indications for RAAP.

## 2. Materials and Methods

A national PPS on HAIs and antimicrobial use was conducted in German acute care hospitals between May and June 2016. Data collection was performed by local hospital staff and in alignment with the PPS methodology described by the European Centre for Disease Prevention and Control (ECDC) [13]. Participation in the survey was possible for all German acute care hospitals, 1951 as of 2016 [14], and on a voluntary basis. Per participating hospital, at least one employee had to undergo data collection training to ensure methodological consistency. The training, as well as the organization of all other aspects of the PPS, was coordinated by the German National Reference Center for Surveillance of Nosocomial Infections. Besides HAIs and data on antimicrobial use, data on selected structural indicators (e.g., hospital type and ownership, number of hospital beds) were collected [13]. All antimicrobial prescriptions that were in effect on the day the survey were recorded. Only patients that had been admitted to the ward before 8 a.m. on the day of survey and that had not yet been discharged were included in the survey. The indication of every antimicrobial prescription was recorded. The PPS protocol distinguished therapeutic from prophylactic (surgical and medical prophylaxis) indications. Therapeutic prescriptions were further separated into treatment for community-acquired infections, hospital-acquired infections, and infections acquired in long-term care facilities. For this analysis, infections acquired in long-term care facilities were regarded as community-acquired infections. Moreover, for therapeutic antimicrobial use, the suspected type of infection was recorded. Data concerning indication of antimicrobial use, and in case of therapeutic prescriptions, the type of infection, had to be retrieved from the patient’s file. If not documented in the patient’s files, data collectors were encouraged to consult the treating physicians in order to obtain missing data.

For the purpose of this analysis, we identified patients for which RAAPs were recorded. RAAPs were defined as the patient simultaneously receiving any of the below-listed combinations:

- PenBLI plus clindamycin;
- PenBLI plus metronidazole;
- PenBLI plus moxifloxacin;
- PenBLI plus carbapenem;
- Carbapenem plus clindamycin;
- Carbapenem plus metronidazole;
- Carbapenem plus moxifloxacin;
- Clindamycin plus metronidazole;
- Clindamycin plus moxifloxacin; and
- Metronidazole plus moxifloxacin.

PenBLI corresponded to the WHO Anatomical Therapeutic Chemical Classification System group J01CR [15]. The decision to classify the above-stated combinations as redundant was made on the basis of the similarity of their therapeutic range [16,17,18]. If a patient received three or more of the above-listed antimicrobials or antimicrobial groups, each RAAP was counted separately, i.e., one patient could have more than one RAAP combination.

Hospitals in Germany are required by the German Protection against Infection Act to collect data on HAIs and antimicrobial use [19]. All data collected in the PPS were anonymized; therefore, ethical approval and informed consent were not required.

## 3. Results

Data from 64,412 patients in 3182 wards of 218 hospitals that participated in the survey were collected. Table 1 summarizes the baseline characteristics of the participating hospitals.

**Figure 1:**
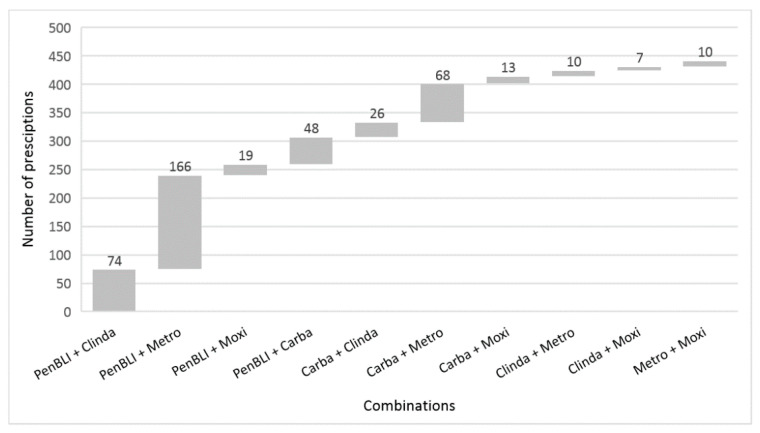
Number of redundant anaerobic antimicrobial prescriptions by the combination of anaerobic antimicrobial agents. Data from 218 hospitals that participated in the point prevalence survey 2016. Abbreviations: Carba—carbapenem; Clinda—clindamycin; Metro—metronidazole; Moxi—moxifloxacin; PenBLI—penicillin/beta-lactamase inhibitor.

The total number of antimicrobials recorded in the survey was 22,086. With regards to patients, 16,688 patients (26%) received at least one antimicrobial, and 4486 patients (7%) received two or more antimicrobials. Of these 4486 patients, 413 (9%) received RAAP combinations. A total of 8541 patients received anaerobic antimicrobials, 413 (5%) of which received RAAP combinations. In total, 441 anaerobic combinations met the above-stated criteria for RAAP. PenBLI plus metronidazole was the most frequently documented anaerobic combination (*N* = 166, 38%), followed by PenBLI plus clindamycin (*N* = 74, 17%) and carbapenem plus metronidazole (*N* = 68, 15%) (Figure 1).

Of all the antimicrobials recorded, 22% of all moxifloxacin prescriptions, 17% of all clindamycin prescriptions, and 16% of all metronidazole prescriptions were part of a redundant anaerobic combination (Table 2).

**Figure 2:**
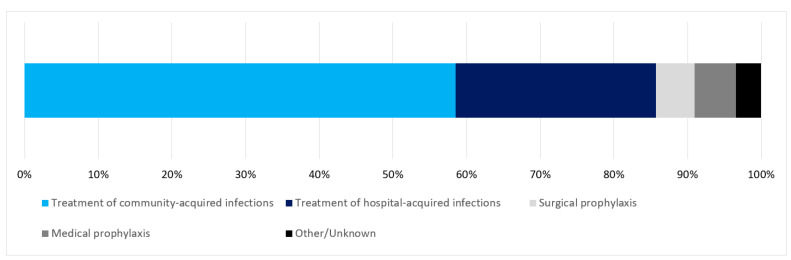
Indications of redundant anaerobic antimicrobial prescriptions as a percentage of all redundant anaerobic antimicrobial prescriptions (*N* = 441). Data from 218 hospitals that participated in the point prevalence survey 2016. All redundant anaerobic antimicrobial prescriptions (*N* = 441); Treatment of community-acquired infections (*N* = 258); Treatment of hospital-acquired infections (*N* = 120); Surgical prophylaxis (*N* = 23); Medical prophylaxis (*N* = 25); Other/Unknown indication (*N* = 15).

The majority of RAAPs were for the treatment of community-acquired (*N* = 258, 59%) and hospital-acquired (*N* = 120, 27%) infections (Figure 2).

Lower respiratory tract infections (LRTIs) (*N* = 77) and skin/soft tissue infections (SSTIs) (*N* = 76) were the most frequently recorded types of infections, both accounting for around 20% of all redundant anaerobic treatments (Table 3).

While SSTIs were the most common type of infection for redundant anaerobic treatment of community-acquired infections (*N* = 57, 22%), LRTIs were the most common type of infection for hospital-acquired infections (*N* = 34, 28%). PenBLI plus metronidazole was the most frequently recorded RAAP combination for community-acquired infections (*N* = 98, 38%), followed by PenBLI plus clindamycin (*N* = 54; 21%). For hospital-acquired infections, PenBLI plus metronidazole (*N* = 40; 33%) and carbapanem plus metronidazole (*N* = 30; 25%) were the most commonly recorded RAAP combinations (Table 4).

A further stratification by the combination of anaerobic antimicrobial agents concerning the type of infection for therapeutic use revealed that PenBLI plus metronidazole (*N* = 25; 32%) and PenBLI plus carbapenem (*N* = 19; 25%) were the most frequently documented RAAPs for LRTIs. For SSTIs, PenBLI plus clindamycin (*N* = 31; 41%) and PenBLI plus metronidazole (*N* = 19; 25%) were the most prevalent RAAP combinations (Table 5).

## 4. Discussion

The PPS 2016 revealed that RAAP is common in German acute care hospitals. Almost one in 10 patients receiving two or more antimicrobials on the day of survey received an RAAP combination.

There is only a limited range of clinical settings, in which treatment with some of the anaerobic combinations included in this analysis warrants a potential benefit for patients. In case of CDI, metronidazole was the medication recommended as first-line treatment at the time of the survey [20,21]. Therefore, some combinations of metronidazole with other antimicrobials, potentially also with other anaerobic antimicrobials, may be attributable to the simultaneous treatment of different medical conditions, where a de-escalation or stop of therapy was not yet possible. Gastrointestinal infections, however, only made up around 13% of all infections treated with redundant anaerobic antimicrobial combinations, and the majority of RAAP combinations containing metronidazole in this study were for non-gastrointestinal infections. Moreover, recent updates of the guidelines for the treatment of CDI have replaced metronidazole with vancomycin per os or fidaxomicin per os [20,21], thereby further decreasing potential indications for redundant anaerobic coverage.

In patients with severe systemic staphylococcal and streptococcal infections, the production of toxins can potentially be inhibited by the addition of clindamycin to the therapeutic regimen [22]. While this may explain some redundant combinations containing clindamycin, it probably only accounts for a fraction of clindamycin use in this study. Since data concerning pathogens was not collected for all antimicrobial prescriptions, this explanation remains speculative.

The most frequent indication for RAAPs was the treatment of community-acquired LRTIs and SSTIs. While anaerobic coverage can be appropriate for the treatment of necrotizing fasciitis, erysipelas and cellulitis are the most common community-acquired SSTIs. For these infections, combinations of anaerobic antimicrobials are generally not recommended [23]. In the case of aspiration pneumonia, anaerobic coverage has historically been included in the treatment by many treating physicians. A recent update of the guideline for community-acquired pneumonia by the American Thoracic Society and Infectious Diseases Society of America, however, does not recommend anaerobic coverage in the calculated treatment strategy [24].

The most frequently documented RAAP combination in this study was PenBLI and metronidazole. In alignment with this result, Huttner et al. also described piperacillin/tazobactam and metronidazole to be the most frequently prescribed anaerobic combination in a study focusing on antimicrobial prescribing behavior in the American Veterans Affair healthcare system [7].

Prudent prescribing of antimicrobials offers considerable potential to decrease the consumption of anti-infective agents. We found that around 22% of all moxifloxacin prescriptions, 17% of all clindamycin prescriptions, and 16% of all metronidazole prescriptions were part of an RAAP combination and thus potentially avoidable. Other studies suggest even higher numbers in this regard [7,25].

The reasons for RAAP have not yet been fully uncovered. A lack of knowledge of the anaerobic coverage in beta lactams, such as PenBLI and carbapenems, is probably one of the reasons for these avoidable prescriptions. Few trials have evaluated the effectiveness of interventions aiming to reduce redundant prescriptions [25]. A recent study from South Korea showed an effective reduction with a pharmacist-led intervention [11], suggesting that a decrease of redundant antimicrobial prescriptions is achievable with relatively simple means.

Various limitations have to be acknowledged when interpreting the data. The analysis conducted for the purpose of this study was a secondary analysis of data collected during the national PPS in Germany in 2016. The primary objective of the PPS was to estimate the prevalence of patients with HAIs and the prevalence of patients receiving antimicrobials. Another important limitation is due to the fact that data collection in participating hospitals was performed by a diverse group of professionals with differences concerning their experience in surveillance and antimicrobial stewardship. The majority of data collectors were non-prescribers of antimicrobials. Therefore, mistakes in documentations due to misinterpretation of the prescriptions represents a potential confounder. To reduce this distorting effect and to ensure a high degree of consistency, all data collectors were systematically trained in the methodology delineated in the ECDC PPS protocol. Furthermore, it is important to acknowledge that no data regarding underlying pathogens in patients receiving antimicrobials for treatment were collected. Therefore, an assessment of whether an RAAP combination was adequate or not remains largely speculative. As participation in the PPS was on a voluntary basis, the data presented in this study were not from a representative sample for the healthcare system in Germany. However, due to the large number of participating hospitals, careful extrapolations to the national level appear justified.

## 5. Conclusions

PPS data can be used in a variety of ways to address aspects of antimicrobial stewardship. This study demonstrates that redundant anaerobic coverage in antimicrobial treatment is common in German acute care hospitals, however, to a lesser extent than reported in other studies. While reasons for this phenomenon are not yet fully understood, improving the prescriber’s knowledge on the anaerobic spectra of beta lactams might be a feasible way to improve the quality of antimicrobial prescriptions in general, and specifically to reduce the frequency of RAAPs. Given the adverse effects of anaerobic antimicrobials on the human gut microbiome, the reduction of RAAPs should be a key target of future antimicrobial stewardship activities. A suitable way for hospitals to reduce RAAPs could be to offer a comprehensive therapy standard for anaerobic infections to prescribing physicians. Furthermore, antimicrobial stewardship teams should place a focus on addressing the issue of RAAP when interacting with colleagues from other medical fields, and on discouraging prescribers with limited experience in the treatment of anaerobic infections to prescribe multiple anaerobic therapeutics simultaneously.

## Figures and Tables

**Table 1:** Baseline characteristics of the 218 hospitals that participated in the point prevalence survey 2016.

Variable	Group/Parameter	Number (Percentage) or Median (Interquartile Range)
Hospital type	Primary care	118 (54.1)
	Secondary care	41 (18.8)
	Tertiary care	36 (16.5)
	Specialized hospital	23 (10.6)
Hospital ownership	Public	103 (47.2)
	Private, not for profit	63 (28.9)
	Private, for profit	31 (14.2)
	Other/Unknown	21 (9.6)
Hospital size	Number of beds	305 (185–541)

The classification for hospital type is based on the definitions of the European Centre for Disease Prevention and Control. Primary care—district hospital, first-referral, has few specialties (e.g., internal medicine, gynecology/obstetrics, pediatrics, general surgery); Secondary care—provincial hospital, has 5 to 10 clinical specialties; Tertiary care—tertiary-level hospital, has highly differentiated specialties, includes university hospitals; Specialized hospital—Solitary clinical specialty.

**Table 2:** Redundant anaerobic prescriptions. Data from 218 hospitals that participated in the point prevalence survey 2016.

Antimicrobial/Antimicrobial Group	All Prescriptions	Redundant Prescriptions (Percentage)
Penicillin/beta-lactamase inhibitor	5119	307 (6.0)
Carbapenems	1369	155 (11.3)
Clindamycin	695	117 (16.8)
Metronidazole	1621	254 (15.7)
Moxifloxacin	227	49 (21.6)

**Table 3:** Types of infections of redundant anaerobic antimicrobial treatments. Data from 218 hospitals that participated in the point prevalence survey 2016.

Type of Infection	Number (Percentage)		
	All Infections	Community-Acquired Infections	Hospital-Acquired Infections
All	378 (100)	258 (100)	120 (100)
Bacteremia	21 (5.6)	17 (6.6)	4 (3.3)
Non-microbiologically confirmed systemic infection	40 (10.6)	21 (8.1)	19 (15.8)
Bone/Joint infection	19 (5.0)	13 (5.0)	6 (5)
Skin/Soft tissue infection	76 (20.1)	57 (22.1)	19 (15.8)
Intra-abdominal infection	32 (8.5)	22 (8.5)	10 (8.3)
Gastrointestinal infection	50 (13.2)	35 (13.6)	15 (12.5)
Lower respiratory tract infection	77 (20.4)	43 (16.7)	34 (28.3)
Urinary tract infection	25 (6.6)	14 (5.4)	11 (9.2)
Other/Not specified	38 (10.1)	36 (14.0)	2 (1.7)

**Table 4:** Indications of redundant anaerobic antimicrobial prescriptions stratified by the combination of anaerobic antimicrobial agents. Data from 218 hospitals that participated in the point prevalence survey 2016.

Combination	Number (Percentage)					
	All	Treatment of CAI	Treatment of HAI	Surgical Prophylaxis	Medical Prophylaxis	Other/Unknown
All	441 (100)	258 (100)	120 (100)	23 (100)	25 (100)	15 (100)
PenBLI + Clinda	74 (16.8)	54 (20.9)	11 (9.2)	5 (21.7)	2 (8)	2 (13.3)
PenBLI + Metro	166 (37.6)	98 (38.0)	40 (33.3)	14 (60.9)	8 (32)	6 (40)
PenBLI + Moxi	19 (4.3)	9 (3.5)	5 (4.2)	0 (0)	4 (16)	1 (6.7)
PenBLI + Carba	48 (10.9)	28 (10.9)	18 (15)	0 (0)	1 (4)	1 (6.7)
Carba + Clinda	26 (5.9)	19 (7.4)	4 (3.3)	2 (8.7)	1 (4)	0 (0)
Carba + Metro	68 (15.4)	26 (10.1)	30 (25)	2 (8.7)	7 (28)	3 (20)
Carba + Moxi	13 (2.9)	7 (2.7)	5 (4.2)	0 (0)	1 (4)	0 (0)
Clinda + Metro	10 (2.7)	7 (2.7)	2 (1.7)	0 (0)	0 (0)	1 (6.7)
Clinda + Moxi	7 (1.6)	3 (1.2)	3 (2.5)	0 (0)	0 (0)	1 (6.7)
Metro + Moxi	10 (2.7)	7 (2.7)	2 (1.7)	0 (0)	1 (4)	0 (0)

Abbreviations: CAI—community-acquired infection; Carba—carbapenem; Clinda—clindamycin; HAI—hospital-acquired infection; Metro—metronidazole; Moxi—moxifloxacin; PenBLI—penicillin/beta-lactamase inhibitor.

**Table 5:** Types of infections of redundant anaerobic antimicrobial treatment stratified by the combination of anaerobic antimicrobial agents. Data from 218 hospitals that participated in the point prevalence survey 2016.

Combination	Number (Percentage)									
	All	BAC	SYS	BJI	SSTI	IA	GI	LRTI	UTI	O/NS
All	378 (100)	21 (100)	40 (100)	19 (100)	76 (100)	32 (100)	50 (100)	77 (100)	25 (100)	38 (100)
PenBLI + Clinda	65 (17.2)	2 (9.5)	3 (7.5)	12 (63.2)	31 (40.8)	0 (0)	1 (2)	5 (6.5)	3 (12)	8 (21.1)
PenBLI + Metro	138 (36.5)	7 (33.3)	12 (30)	1 (5.3)	19 (25)	18 (56.3)	34 (68)	25 (32.5)	11 (44)	11 (28.9)
PenBLI + Moxi	14 (3.7)	3 (14.3)	1 (2.5)	0 (0)	2 (2.6)	0 (0)	1 (2)	6 (7.8)	0 (0)	1 (2.6)
PenBLI + Carba	46 (12.2)	1 (4.8)	5 (12.5)	1 (5.3)	5 (6.6)	6 (18.8)	2 (4)	19 (24.7)	5 (20)	2 (5.3)
Carba + Clinda	23 (6.1)	1 (4.8)	4 (10)	2 (10.5)	8 (10.5)	0 (0)	0 (0)	5 (6.5)	0 (0)	3 (7.9)
Carba + Metro	56 (14.8)	5 (23.8)	13 (32.5)	0 (0)	2 (2.6)	7 (21.9)	8 (16)	9 (11.7)	4 (16)	8 (21.1)
Carba + Moxi	12 (3.2)	0 (0)	1 (2.5)	0 (0)	0 (0)	1 (3.2)	2 (4)	4 (5.2)	2 (8)	2 (5.3)
Clinda + Metro	9 (2.4)	0 (0)	1 (2.5)	2 (10.5)	3 (3.9)	0 (0)	1 (2)	0 (0)	0 (0)	2 (5.3)
Clinda + Moxi	6 (1.6)	0 (0)	0 (0)	1 (5.3)	3 (3.9)	0 (0)	0 (0)	2 (2.6)	0 (0)	0 (0)
Metro + Moxi	9 (2.4)	2 (9.5)	0 (0)	0 (0)	3 (3.9)	0 (0)	1 (2)	2 (2.6)	0 (0)	1 (2.6)

Abbreviations: BAC—bacteremia; BJI—bone/joint infection; Carba—carbapenem; Clinda—clindamycin; GI—gastrointestinal infection; IA—intraabdominal infection; LRTI—lower respiratory tract infection; Metro—metronidazole; Moxi—moxifloxacin; O/NS—other/not specified; PenBLI—penicillin/beta-lactamase inhibitor; SSTI—skin/soft tissue infection; SYS—non-microbiologically confirmed systemic infection; UTI—urinary tract infection.
